# The Principles and Practice of Midwifery

**Published:** 1848-04

**Authors:** 


### Bibliographical Notices.

*The Principles and Practice of Midwifery.* By David H. Tucker, M. D., Professor of the Principles and Practice of Medicine, &c. Philadelphia: Lindsay & Blakiston, 1848.

*Elements of General Pathology.* By Alfred Stille, M. D., Lectures on Pathology and the Practice of Medicine, in the Philadelphia Medical Association, &c. Philadelphia: Lindsay & Blakiston, 1848.

The two above named works are number one and two of the "Medical Practitioners, and Students' Library," comprising works on every department of medicine, making a library of reference for the practitioner, or a complete set of text books for the student, which the enterprising publishers, Messrs. Lindsay & Blakiston, propose to present to the profession in a cheap form—thus, presenting to the dentist an opportunity by becoming a subscriber, at a small expense, of procuring a medical library.

Without a wish to offend, we must express what has long been observed, a blamable deficiency of medical knowledge in the dental profession. There are too many of its members who practice the profession, relying on mechanical principles, and unacquainted with medical facts and doctrines.

There have been but few works if any, in the English language, that have devoted *as much* space to the subject of general pathology in its proper sense as was necessary for the proper advancement of the student, or the wish of the practitioner. One of the objects of Dr. Stille, in this work on general pathology is to fill this vacancy in medical literature. He makes, in the introduction to the work, the following observation, which is as applicable to the dental as to the medical profession, that "he is convinced that among the most serious defects in the present system of medical education, are an almost total neglect of logical analysis, and a tendency, where generalization is at all encouraged, to speculate on fanciful analogies, rather than to extract truth from fact. How fatal these influences must be to a successful study of disease, and to that confidence in medicine which experience imparts to the philosophical physician, is too apparent to require illustration. The author is persuaded that the view of medicine since which it is the province of general pathology to afford, is eminently adapted to correct these evils, by encouraging a more healthful discipline, and infusing a more hopeful spirit among those who are preparing for medical practice."

We feel that we cannot render the members of the dental profession a greater service, than to urge them to embrace this opportunity of procuring a medical library at a trifling expense by adding their names as subscribers to this series of medical publications, which can be received through the mail at periodical postage. The whole series can be had for $1 per vol. or even at that price by subscribing for only five numbers.

The above works will be succeeded by the following named works, at intervals of two months or less, viz. Diseases of Children, by JOHN F. MEYS, M. D., Lecturer on Obstetrics and Diseases of Children, in the Philadelphia Medical Association.

Clinical Medicine or Internal Pathology and Therapeutics by MERIDETH CLYMER, M. D., late Professor of the Principles and Practice of Medicine, in the Franklin Medical College, Philadelphia.

Minor Surgery, by EDWARD HARTSHORNE, M. D., Lecturer on Legal Medicine, in the Philadelphia Medical Association.

General or Microscopic Anatomy, by FRANCIS G. SMITH, M. D., Lecturer on Physiology in the Philadelphia Medical Association.

Special Anatomy, by JOHN NEAL, Lecturer on Anatomy, and Demonstrator in the University of Pennsylvania.

Physiology, by FRANCIS G. SMITH, M. D., Lecturer on Physiology in the Philadelphia Medical Association.

Medical Chemistry, by ROBERT BRIDGES, M. D., Professor of Chemistry in the Philadelphia College of Pharmacy, &c.

Materia Medica and Therapeutics, by FRANCIS WEST, M. D., Lecturer on Materia Medica and Therapeutics in the Philadelphia Medical Association.

Clinical Surgery, by EDWARD HARTSHORNE, M. D., Lecturer on Legal Medicine in the Philadelphia Medical Association.

The American Dissector, prepared on a new plan by J. W. ALLEN, M. D., Demonstrator of Anatomy, &c.

The names of the Authors of the above works, and the reputation of the publishers are a sufficient guarantee that the works will be all that the profession have a right to expect.

